# CIITA and Its Dual Roles in MHC Gene Transcription

**DOI:** 10.3389/fimmu.2013.00476

**Published:** 2013-12-20

**Authors:** Ballachanda N. Devaiah, Dinah S. Singer

**Affiliations:** ^1^Experimental Immunology Branch, National Cancer Institute, NIH, Bethesda, MD, USA

**Keywords:** CIITA, MHC transcription, NLR/CATERPILLER proteins, enhanceosome, TAF1, general transcription factors

## Abstract

Class II transactivator (CIITA) is a transcriptional coactivator that regulates γ-interferon-activated transcription of Major Histocompatibility Complex (MHC) class I and II genes. As such, it plays a critical role in immune responses: CIITA deficiency results in aberrant MHC gene expression and consequently in autoimmune diseases such as Type II bare lymphocyte syndrome. Although CIITA does not bind DNA directly, it regulates MHC transcription in two distinct ways – as a transcriptional activator and as a general transcription factor. As an activator, CIITA nucleates an enhanceosome consisting of the DNA binding transcription factors RFX, cyclic AMP response element binding protein, and NF-Y. As a general transcription factor, CIITA functionally replaces the TFIID component, TAF1. Like TAF1, CIITA possesses acetyltransferase (AT) and kinase activities, both of which contribute to proper transcription of MHC class I and II genes. The substrate specificity and regulation of the CIITA AT and kinase activities also parallel those of TAF1. In addition, CIITA is tightly regulated by its various regulatory domains that undergo phosphorylation and influence its targeted localization. Thus, a complex picture of the mechanisms regulating CIITA function is emerging suggesting that CIITA has dual roles in transcriptional regulation which are summarized in this review.

The class II transactivator (CIITA) is a master regulator of major histocompatibility complex (MHC) gene expression. It induces *de novo* transcription of MHC class II genes and enhances constitutive MHC class I gene expression. The role of CIITA in regulating MHC gene transcription is well established: CIITA deficiency or aberrant expression is linked to the Type II bare lymphocyte syndrome and to cancer, respectively (1, 2). Although CIITA has been primarily characterized as a transcriptional regulator of MHC genes, it also regulates transcription of over 60 immunologically important genes, including interleukin 4 (IL-4), IL-10, and a variety of thyroid-specific genes (3, 4). Despite the important role of CIITA in regulating expression of these genes, the actual mechanisms by which it functions are still being unraveled. Early studies established that CIITA is a coactivator that nucleates the formation of an enhanceosome with transcription factors binding to enhancer elements in the upstream regions of the MHC genes (5, 6). In addition, more recent studies from our lab have demonstrated that it also functions as a component of the basal transcriptional machinery (7–9). Here we review the two distinct mechanisms by which CIITA regulates transcription of MHC class I and II genes and speculate on how these activities may be interconnected.

## CIITA Belongs to the Family of NLR/CATERPILLER Proteins

The members of the NLR/CATERPILLER family of proteins are defined by their structures which include both a nucleotide binding domain (NBD) and a C-terminal leucine rich region (LRR) (10). Of the nearly two dozen genes within the family, many encode proteins that mediate inflammatory responses and whose aberrant expression has been correlated with a variety of diseases (11). Of these, only CIITA is a critical mediator of adaptive immunity. Constitutive expression of CIITA is limited to antigen presenting cells. However, γ-interferon exposure induces *de novo* CIITA expression in most cell types (12, 13). Its activity is known to be modulated by several posttranslational modifications including phosphorylation, ubiquitination, acetylation, and deacetylation (14). CIITA can also self-associate and oligomerize (15, 16). CIITA activity is further modulated by its cellular localization: the GTP binding domain (GBD) of CIITA regulates its shuttling between the nucleus and cytoplasm (17, 18).

Class II transactivator consists of a series of regulatory domains that include an activation domain (AD), an acetyltransferase (AT) domain, a proline/serine/threonine (PST) domain, a GBD and finally, the canonical LRR domain common to all NLR proteins. The CIITA AD domain binds to general transcription factors and the CREB-binding protein (CBP), leading to activation of the MHC class II promoter and repression of the IL-4 promoter (19, 20). This domain also partially overlaps the region required for the AT activity of CIITA (7). The role of the PST domain, which while essential for CIITA function, remains unknown (21). The LRR domain, which interacts with the GBD, is known to play an important role in CIITA movement into the nucleus and in regulating its transactivation function (15, 21, 22). The GBD domain, which is perhaps the best studied among the CIITA domains, has been shown to be the site of interaction of several DNA binding transactivators (23). The GBD regulates translocation of CIITA: mutation or deletion of the GBD results in increased nuclear export, suggesting that it is a negative regulator of CIITA nuclear export (18). Two nuclear localization signals have been mapped to the N-terminal domain, as well as an additional one in the C-terminus (18). CIITA also contains two LxxLL motifs which are crucial for the transactivation function and self-association of CIITA (24). In addition to these multifunctional domains, CIITA also contains two degrons in the AD and PST domains respectively. These degrons signal the rapid degradation of CIITA through the ubiquitin-proteasome pathway, and are responsible for the instability and short half-life of CIITA (~30 min) (25). CIITA function is also regulated through its oligomerization, which has been shown to be mediated by amino acids 253–410 (16). Each of the domains and regions listed above are also the targets of multiple post translational modifications which control their function (14). The expression of the CIITA gene itself is regulated by a complex mechanism involving four promoters and five enhancers that combine to form a dynamic chromatin structure (26, 27). Thus, CIITA is an extremely complex, unstable, and short lived protein, suggesting that its presence and function are tightly regulated.

## CIITA Functions as a Transactivator by Nucleating an Enhanceosome

The role of CIITA as a central component of an enhanceosome has been characterized primarily for the MHC genes. MHC class II genes are transcriptionally controlled via conserved *cis*-acting elements in their promoters. These elements, named the W/S, X, X2, and Y boxes interact with specific *trans*-activating DNA binding factors to regulate MHC transcription either positively or negatively (28). The DNA binding factors involved, namely RFX (a hetero-multimer consisting of subunits RFX5, RFX-ANK, and RFX-AP), cyclic AMP response element binding protein (CREB), and NF-Y (A, B, and C), bind directly to the X, X2, and Y boxes respectively. These factors are constitutively expressed but their binding to the class II gene is not sufficient to support expression (29). CIITA interacts with each of these DNA binding factors (30). Those interactions depend on distinct structural domains within CIITA. CIITA transactivator function is dependent on the AD, PST, GBD, and LRR domains within its primary protein structure. RFX-ANK and NF-YC bind to the N-terminal acidic AD, whereas the remaining *trans*-activating factors interact with the GBD (23). The interaction of CIITA with the DNA-bound transcription factors serves to form a transcriptionally active complex, or enhanceosome (5). Importantly, the cognate DNA binding sites are spaced in a manner that supports the formation of an enhanceosome complex anchored by CIITA (31).

Within the enhanceosome, CIITA functions in the recruitment of various histone modifying enzymes, both activating and repressive. During MHC gene activation, CIITA recruits histone modifying enzymes such as the ATs p300, CBP, and the p300/CBP-associated factor (PCAF), as well as the methyltransferase CARM1 which function to support active transcription (32). In contrast, during MHC gene silencing, CIITA and RFX recruit and bind histone deacetylases HDAC1, HDAC2, and HDAC4 (31). CIITA also interacts with chromatin remodeling factors, such as BRG1 (33), and other coactivactors such as SRC-1 (32). Thus, while CIITA does not directly bind DNA, it serves to nucleate and coordinate the various transcription factors and chromatin modifying enzymes that are necessary to support transcription of the class I and II genes.

The concept of a CIITA-nucleated enhanceosome fits well with the regulation of MHC class I and II transcription, and is supported by considerable circumstantial evidence. Such evidence includes the demonstration of direct interaction between CIITA and the other components of the predicted enhanceosome (5). However, CIITA also transactivates many genes that may not possess the *cis*-elements and *trans*-factors that are found on MHC genes (3). This raises the possibility that CIITA may also have a more direct function in transcription besides nucleating an enhanceosome. Indeed, as will be discussed below, CIITA functions as a component of the basal transcription machinery.

## CIITA Functions as a General Transcription Factor and Functional Homolog of TAF1

The basal transcriptional machinery requires the assembly on the core promoter of a pre-initiation complex (PIC) that plays a central role in regulating transcription initiation. PIC assembly is initiated by the binding of the TFIID general transcription factor complex – composed of the TATA-binding protein (TBP) and a set of TBP-associated factors (TAFs) – to the core promoter (34). The largest factor in the TFIID complex, TAF1, has AT and kinase activities both of which are essential for transcription initiation (35, 36). In early studies from our lab, it was demonstrated that constitutive transcription of MHC class I genes depends on the AT activity of TAF1: MHC class I expression was abrogated at restrictive temperatures in TAF1 temperature-sensitive mutants. In contrast, CIITA-activated transcription of MHC genes was unaffected under these conditions, and thus independent of TAF1 function (37). This finding led to our discovery that CIITA, like TAF1, has an intrinsic AT activity and can bypass the requirement for TAF1 (7). Based on these findings, we proposed that CIITA functions as a general transcription factor that can substitute for TAF1 function during γ-interferon-activated MHC transcription (38).

Consistent with the model that CIITA is a general transcription factor that assembles a TFIID-like complex, CIITA is known to recruit and directly interact with components of the TFIID complex, including TBP, TAF6, and TAF9, as well as PTEFb and TFIIB which are components of the PIC (39, 40). Further supporting this model, we found that CIITA interacts with the TFIID component TAF7 (8) which acts as a check-point regulator of constitutive class I transcription initiation by inhibiting TAF1 AT activity (41). TAF7 binds directly to the region encompassing the AT domain of CIITA. Importantly, the binding of TAF7 to CIITA *in vitro* inhibits both its AT activity and transcription. *In vivo* knock-down of TAF7 resulted in a significant increase in CIITA-activated MHC class I gene expression (8). Taken together, these findings suggested that CIITA is a functional homolog of TAF1.

Further evidence for the parallels between CIITA and TAF1 came from the finding that CIITA, like TAF1, is a kinase (9). In the canonical TFIID complex, TAF1 is associated with and inhibited by TAF7 until PIC assembly is complete. Transcription initiation requires the release of TAF7 from TAF1, thereby revealing TAF1’s essential AT activity. The release is mediated by TAF1 autophosphorylation by its intrinsic kinase activity, which is essential for initiating transcription (42). Interestingly, although TAF1 also phosphorylates TAF7, this does not cause the release of TAF7 from the TFIID complex. Rather, it modulates the subsequent regulation of TFIIH, BRD4, and PTEFb transcription factors by TAF7 (43, 44). The finding that CIITA AT activity bypasses the requirement for TAF1 during activated MHC class I transcription and that TAF7 inhibits this activity suggested that CIITA also might have a kinase activity responsible for dissociating TAF7. Indeed, we recently found that CIITA has intrinsic kinase activity (9). CIITA, like TAF1, autophosphorylates. This autophosphorylation prevents the binding of TAF7 which would otherwise inhibit its AT activity. CIITA phosphorylates TAF7, although at sites distinct from those phosphorylated by TAF1 (9). It remains to be determined whether TAF7 phosphorylation by CIITA, like by TAF1, modulates the subsequent regulation of TFIIH and PTEFb by TAF7.

Similar to TAF1, the AT activity of CIITA was identified by its ability to acetylate histones *in vitro* (7). However, the actual *in vivo* substrate(s) for both CIITA and TAF1 remain to be defined. This leaves open the possibility that CIITA acetylates any of its numerous interacting partners *in vivo*. Additionally, the AT activity of CIITA is modulated by its own GBD; the binding of GTP at this site increases AT activity and nuclear localization of CIITA (7). Acetylation of CIITA on its N-terminal nuclear localization domain by PCAF is also known to be a signal for its nuclear localization (45). However, the loss of its AT domain does not affect CIITA’s nuclear localization (18). Thus, while it is established that the AT activity of CIITA is undoubtedly required for its function in transcriptional regulation, many questions remain regarding the targets of this activity, the complexity of its regulation both directly by trans factors and indirectly by other regulatory domains on CIITA.

Class II transactivator function is also regulated by phosphorylation, as has been well documented (16, 46, 47). CIITA is known to be phosphorylated by PKA, PKC, GSK3, CK1, ERK1/2, and CKII kinases at various sites spanning its AT, PST, and LRR domains (14). These phosphorylation events are known to regulate its transactivation function, nuclear localization, oligomerization, and its ability to interact with DNA binding transactivators. The newly discovered kinase activity of CIITA adds an additional layer of complexity to the functional mechanisms by which CIITA operates. The kinase activity of CIITA and its ability to autophosphorylate are thus likely to have significant ramifications on its function. Indeed, we found that autophosphorylation of CIITA enhances its AT activity *in vitro*, suggesting that the two enzymatic activities of CIITA are interconnected. In contrast to the AT activity of CIITA, at least three independent substrates for CIITA kinase activity have been found thus far including TAF7, Histone H2B, and the TFIIF component RAP74 (9). While CIITA autophosphorylation regulates its ability to interact with TAF7 and consequently its AT activity, the purpose of TAF7 phosphorylation by CIITA is yet to be discovered. By analogy with TAF1 (44), we speculate that it modulates TAF7 binding to its downstream targets, BRD4, PTEFb, and TFIIH. RAP74 phosphorylation by TAF1 is thought to help in coordinating the functions of different components of the pre-initiation complex (36). Whether CIITA phosphorylation of RAP74 serves the same purpose remains to be seen. Similarly, the phosphorylation of Histone H2B at Ser36 has been demonstrated to play a significant role in regulating transcription during cell cycle progression and stress response (48, 49). Therefore, the ability of CIITA to phosphorylate Histone H2B Ser36 (9) supports the idea that it has a role beyond its known function in regulating MHC genes. The substrate specificities of CIITA kinase activity, although very similar to those of TAF1, are distinct. CIITA phosphorylates TAF7 at a different site and phosphorylates all histones unlike TAF1 (9). We speculate that the distinct nature of CIITA kinase activity suggests that it may have other substrates, possibly in the enhanceosome complex that it nucleates.

## Integrating the Dual Functions of CIITA: A Model

**Figure 1:**
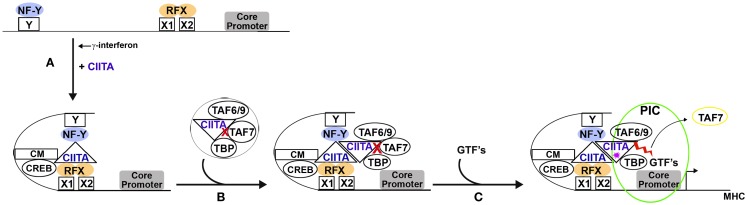
**Proposed model for the dual roles of CIITA in MHC gene transcription**. **(A)** The induction of CIITA, in response to γ-interferon, leads to the assembly of an enhanceosome through CIITA’s interactions with CREB and the NF-Y and RFX transactivators associated with the X and Y box DNA elements, respectively. In addition, CIITA interacts with chromatin modifying (CM) enzymes, contributing to chromatin remodeling. **(B)** Independently, CIITA recruits several components of the canonical TFIID complex, namely TAF6/9, TAF7, and TBP. The CIITA in the TFIID-like complex is recruited to and dimerizes with the CIITA present at the enhanceosome. TAF7 keeps the acetyltransferase activity of CIITA in check during this process (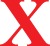). **(C)** The arrival of other general transcription factors (GTFs) completes assembly of the pre-initiation complex, leading to autophosphorylation of CIITA (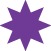). CIITA autophosphorylation triggers the release of TAF7 and reveals the AT activity of CIITA (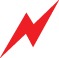), allowing transcription to initiate.

Although CIITA has no obvious structural homology with TAF1, it has remarkable functional parallels. Like TAF1, CIITA associates with general transcription factors to form a TFIID-like complex (8, 39, 40). Both CIITA and TAF1 have AT activity that is essential for transcription (7, 35, 41); both AT activities are regulated by TAF7 (8, 41). CIITA and TAF1 both have kinase activity that results in autophosphorylation leading to TAF7 dissociation (9, 36, 42). Taken together, these findings demonstrate that CIITA functions as a general transcription factor and functional homolog of TAF1, independent of its role as a coactivator that nucleates an enhanceosome. The dual functionality of CIITA, as a coactivator and a general transcription factor, leads to the question of how these two disparate functions are coordinated during MHC gene regulation. We propose the following model (Figure 1).

Following induction by γ-interferon, CIITA nucleates the formation of an enhanceosome through its interaction with transactivators that are constitutively bound to conserved DNA elements within the extended promoters of MHC class I and II (Figure 1A). We speculate that within the enhanceosome, CIITA may participate in chromatin remodeling, along with the histone modifying enzymes it recruits to the enhanceosome. Indeed, CIITA efficiently acetylates histones H3 and H4 (7). The acetylations may serve to maintain the MHC class I- and II-associated chromatin in a transcriptionally active conformation. Alternatively, within the enhanceosome, CIITA may acetylate any one of its interacting partners: the binding site on CIITA for many of the CIITA interacting partners in the MHC II enhanceosome (e.g., RFX-ANK and NF-YC) maps to the N-terminal α-helical acidic domain between amino acids 58 and 94 (23), which is immediately adjacent to the CIITA AT domain between amino acids 94 and 132 (7). Whether these factors are substrates of the AT activity or whether their binding to CIITA within the context of the enhanceosome activates or represses AT activity remain intriguing questions. CIITA also efficiently phosphorylates all four histones (9). Phosphorylation of H2B by CIITA has been mapped to Ser36 (9), an event that leads to increased transcription and survival during cell stress (49). Thus it is possible that CIITA’s enzymatic activities contribute to its coactivator function.

We further speculate that the TFIID-like complex assembled by CIITA is recruited to the promoters of MHC class I and II genes by the CIITA-nucleated enhanceosome through dimerization of the CIITA molecules contained within each complex (Figure 1B). According to this model, following nucleation by CIITA, the enhanceosome would interact with the TFIID-like CIITA complex and deliver it to the downstream core promoter. The binding of the TFIID-like CIITA complex to the core promoter would trigger the assembly of a PIC. Once PIC assembly is complete, CIITA would autophosphorylate, releasing TAF7 from the TFIID-like complex. The release of TAF7 would relieve the inhibition of the AT activity associated with the CIITA in the TFIID-like complex. This AT activity, like that of TAF1, is required for transcription initiation.

As noted above, CIITA also activates transcription of non-MHC genes that do not support formation of enhanceosomes comprised of NF-Y and RFX (3). We speculate that these promoters recruit CIITA to the upstream regulatory regions of promoters through its binding to a variety of other transcription factors. This CIITA, by dimerization with CIITA in the TFIID-like complex is then able to deliver it to the core promoter. Future experiments will test this model.

The fact that CIITA is one of a family of NLR/CATERPILLER proteins also raises the possibility that other members of the family may have similar bi-functionality. Indeed, NLRC5, another NLR family member, regulates MHC class I gene transcription (50). NLRC5 domain structure is similar to that of CIITA; it has been proposed to assemble an enhanceosome on MHC class I promoters. It will be of interest to determine whether NLRC5 has enzymatic activities and dual roles in transcription analogous to those of CIITA.

## Conclusion

Class II transactivator regulates transcription of both MHC and non-MHC genes through its multiplicity of functions. It functions as a transcriptional transactivator in assembling an enhanceosome with transcription factors bound to the distal promoters of MHC genes and as a general transcription factor and functional homolog of TAF1 associated with the core promoter. While it is possible that these CIITA functions are independent, the cross-regulation of the AT and kinase activities of CIITA suggests that they are interconnected. Thus, we speculate that CIITA initially assembles an enhanceosome with DNA-bound transactivators, first recruiting histone modifying enzymes and then a TFIID-like complex containing CIITA that nucleates assembly of the PIC. Within the PIC, CIITA functionally replaces TAF1 to initiate transcription. As other substrates for the enzymatic activities of CIITA are discovered in the future, it is likely that the complexity of CIITA function will further increase. Which genes CIITA activates as a basal transcription factor, and if this is determined by the presence or absence of upstream nucleation sites for an enhanceosome, will also be subjects for future research.

## Conflict of Interest Statement

The authors declare that the research was conducted in the absence of any commercial or financial relationships that could be construed as a potential conflict of interest.
